# Interventions to reduce cancer screening inequities: the perspective and role of patients, advocacy groups, and empowerment organizations

**DOI:** 10.1186/s12939-023-01841-6

**Published:** 2023-01-27

**Authors:** Afua Richardson-Parry, Carole Baas, Shaantanu Donde, Bianca Ferraiolo, Maimah Karmo, Zorana Maravic, Lars Münter, Ignacio Ricci-Cabello, Mitchell Silva, Stacey Tinianov, Jose M. Valderas, Seth Woodruff, Joris van Vugt

**Affiliations:** 1 Viatris Global Healthcare UK, Building 4, Trident Place, Mosquito Way, Hatfield, London, AL10 9UL UK; 2 Alamo Breast Cancer Foundation, 909 Midland Creek Drive, Southlake, TX 76092 USA; 3 Viatris Global Healthcare, Building 4, Trident Place, Mosquito Way, Hatfield, London, AL10 9UL UK; 4 Cittadinanzattiva - Active Citizenship Network, Rue Philippe Le Bon 46, 1000 Brussels, Belgium; 5 Tigerlily Foundation, 42020 Village Center Plaza, #120-156, Stone Ridge, 20105 USA; 6 Digestive Cancers Europe, Rue de la Loi 235/27, 1040 Brussels, Belgium; 7 Danish Committee for Health Education, Classensgade 71, 5, 2100 Copenhagen, Denmark; 8 Balearic Islands Health Research Institute (IdISBa) and CIBER de Epidemiología y Salud Pública (CIBERESP), C/ Escola Graduada 3, 07002 Palma, Balearic Islands Spain; 9 Esperity, Clos Chapelle-aux-Champs 30, 1200 Brussels, Belgium; 10 Advocates for Collaborative Education, 824 Windsor Street, Santa Cruz, CA 95062 USA; 11 National University of Singapore, 1E Kent Ridge Road, NUHS Tower Block, Singapore, 119228 Singapore; 12 Viatris, 235 E42nd Street, New York, 10017 USA; 13 Viatris, Aalsterweg 172, 5644 RH Eindhoven, The Netherlands

**Keywords:** Health equity, Colorectal cancer, Breast cancer, Cervical cancer, Oncology, Mammography, Pap test, Race, Socioeconomic status

## Abstract

**Background:**

Health inequities lead to low rates of cancer screening in certain populations, such as low-income and ethnic minority groups. Different interventions to address this have been developed with mixed results. However, interventions are not always developed in collaboration with the people they target. The aim of our article is to present the viewpoint of patients, survivors, advocates, and lay persons on interventions to increase cancer screening from a health inequity perspective.

**Methods:**

We prepared talking points to guide discussions between coauthors, who included representatives from nine patient and survivor advocacy groups, organizations working for citizen/patient empowerment, and health equity experts. Perspectives and opinions were first collected through video conferencing meetings and a first draft of the paper was prepared. All authors, read through, revised, and discussed the contents to reach an agreement on the final perspectives to be presented.

**Results:**

Several themes were identified: it is important to not view screening as a discrete event; barriers underlying an individual’s access and willingness to undergo screening span across a continuum; individually tailored interventions are likely to be more effective than a one-size fits-all approach because they may better accommodate the person’s personal beliefs, knowledge, behaviors, and preferences; targeting people who are unknown to medical services and largely unreachable is a major challenge; including professional patient advocacy groups and relevant lay persons in the cocreation of interventions at all stages of design, implementation, and evaluation is essential along with relevant stakeholders (healthcare professionals, researchers, local government and community organizations etc).

**Conclusions:**

Interventions to address cancer screening inequity currently do not adequately solve the issue, especially from the viewpoint of patients, survivors, and lay persons. Several core pathways should be focused on when designing and implementing interventions: advancing individually tailored interventions; digital tools and social media; peer-based approaches; empowerment; addressing policy and system barriers; better design of interventions; and collaboration, including the involvement of patients and patient advocacy organizations.

## Introduction

Health inequity has been broadly defined as “systematic differences in the health status of different population groups” (https://www.who.int/news-room/facts-in-pictures/detail/health-inequities-and-their-causes), and refers to unfair and avoidable differences that could arise from poor governance, corruption, or cultural exclusion [1], whereas the term health inequality relates to uneven distributions of health or health resources due to genetic or other factors or lack of resources [1]. It is well established that there are many sources of health inequity in oncology, from differences in cancer-related mortality according to sex, race, and socioeconomic factors [2–4], inequities in time to diagnosis for some cancer types [5], and disparities in access to treatment and care [3, 6, 7], to name just a few. Studies show large differences in cancer screening uptake according to several factors, including sex, socioeconomic status, and ethnicity as well as being part of a vulnerable or minority group [8–18]. These associations confirm the inverse care law [19], by which the burden of health needs is inversely associated with access to the relevant health services.

Screening is a vital public health strategy for the early identification of cervical, prostate, breast, and colorectal cancers as it allows for more timely access to treatment and care, which may also impact survival and long-term clinical outcomes. For example, if every woman regularly attended cervical cancer screening, 83% of cervical cancer deaths could be prevented [20]. Further, colorectal cancer [21] and breast cancer [22, 23] mortality can be reduced with screening.

Interventions that aim to increase screening uptake are an important approach for tackling inequity. The reasons underlying differences in cancer screening uptake are multifaceted [14] and cover a wide range of themes from perceived racial discrimination and racial residential segregation to stigma and sociodemographic and cultural factors, as well as medical mistrust and perceived susceptibility, benefits, and barriers [24–28]. Therefore, attempts to address health inequities and increase screening uptake can be multifaceted. Indeed, behavior change is complex; an international consensus identified 93 distinct behavioral change techniques [29], for example active components of behavior change interventions can include financial reward, threat, habit reversal, social support, shaping knowledge, comparative imagining of future outcomes, goal-setting etc. There are many randomized control trials in the scientific literature that compare screening uptake in participants undergoing interventions compared to usual care [30–47]. Interventions can range from screening reminders and alerts to the use of lay health workers or healthcare professionals to increase health literacy and address barriers through individual or group counselling, to providing culturally tailored print or video materials, or even giving financial rewards to people for completing screening [33, 36, 42, 43, 48–54]. Some interventions also utilize interactive, individually-tailored, digital technologies [46, 55, 56]. The success of trials in terms of increasing screening uptake are mixed. Further, interventions that are conducted in controlled research environments might not be transferable to real-world setting or on a large scale, though pragmatic randomized control trials may help with this. On the other hand, community-based interventions run by advocacy groups and charities might not be able to accurately evaluate the efficacy of their programs unless it is assessed using a randomized controlled research approach. Many research trials are developed by sponsors, researchers, healthcare systems, or healthcare professionals without direct involvement of patients, citizens, or the people they are targeting and, thus, little is known about their wishes and preferences. It is important to engage patients, the public, citizens, advocacy and empowerment groups, researchers and healthcare workers, and other relevant players, to collaborate on designing and assessing the impact and success of interventions.

The aim of the current article was to gather the perspectives of layperson stakeholders on interventions to increase cancer screening completion from a health inequity perspective. Specifically, we aim to present the opinion of patients, survivors, and patient advocacy and empowerment groups, and research experts on the sources of cancer screening inequity, barriers to screening uptake, and the benefits and limitations of current interventions to reduce cancer screening inequities. A further objective was to explore the role of patients, the public, and advocacy groups in the development and evaluation of such interventions.

## Method

The current paper presents the perspectives and opinions of the authors, which include patients, representatives from citizen and patient empowerment and advocacy groups, and research experts.. The authors have experience in a wide range of oncological areas (digestive cancers, breast cancer etc.) and included individuals from organizations working with health education and citizen/patient empowerment (see author list and affiliations). We focused mostly on breast, cervical, and colorectal cancers because they usually have wide-spread public health screening programs that target the whole population within a certain age group (ie, as opposed to screening for lung cancer which is usually done in specific high-risk groups such as heavy smokers). We prepared talking points (Table 1) that would be used to guide discussions between coauthors based on examples of interventions from the scientific literature. Perspectives and opinions were first collected through video conferencing meetings and a first draft of the paper was prepared. All authors, read through, revised and discussed the contents in order to reach an agreement on the final perspectives to be presented. All results reflect the opinions and perspective of the authors.

**Table 1:** Semi-structured questions used to guide discussion

- Which groups do you think need to be prioritized due to health inequities in cancer screening?	
- What are the current barriers to cancer screening uptake in the different health inequity groups?	
- Do you think that the current scientific literature accurately reflects ongoing initiatives to increase screening uptake in low-uptake groups?	
- Are you aware of any interventions currently used for your patients of interest?	
- Which types of interventions do you think will be most applicable to your patients?	
- What barriers are there that would prevent such an intervention being implemented?	
- Which pros and cons do you envisage with these interventions?	
- What are patient preferences for screening and interventions to increase screening uptake?	
- Which components do you believe are the most essential in the design of an intervention?	
- How can patients and lay persons help to develop effective interventions that have positive outcomes?	

## Results

### Perspectives on sources of cancer screening inequities and current barriers to cancer screening uptake

We identified several areas of cancer screening inequity, including social, economic, and cognitive inequity, as well as differences in health literacy. Of note, it is important to highlight differences between the USA and Europe, particularly due to the lack of universal healthcare coverage in the former. Individuals from the US-based patient advocacy groups described important barriers to screening that were linked to wider issues of both inequality and inequity, including systemic racisms and historical and current oppression. All authors emphasized the importance of recognizing that health inequity is multifaceted and, although interventions in the scientific literature often focus on racial, economic, or cultural inequity, there are also inequities that arise from social and cognitive aspects, such as people who are alone without socially supportive environments, who may need people to support their decision-making or accompany them to screening, or older people with low digital literacy who have difficulties with digital booking systems.

The importance of not viewing cancer screening as a discrete event was discussed; instead, it spans across a continuum in terms of the barriers that underlie an individual’s access and willingness to undergo screening. For example, a person’s decision to undergo screening may be influenced by their cultural and personal beliefs, past and current life experiences, events that have happened at different points in time, as well as current practical and logistical factors, and potential concerns for the future. One author proposed that health and illness could be viewed as a ladder, with a person climbing up each rung, and fear of what might be at the top could prevent them from climbing upwards. With each step, the individual needs to overcome a new barrier. Another gave concrete examples from the USA of how patients had delayed screening because they had no financial or logistical means to miss a work shift or did not have childcare. Individuals have other priorities than their health and may not want to risk or sacrifice their job for health screening. In other cases, the fear of the long-term consequences of being ill and concerns about coping with such an event logistically, financially, and emotionally can prevent them from completing screening. Addressing this avoidance and fear of being diagnosed with cancer can only be done by considering what those long-term fears entail. Many authors were skeptical about interventions that focus only on increasing screening uptake without addressing the subsequent stages of health inequity that occur if a person has a positive screening result, in terms of speed of diagnosis, as well as access to timely care, treatment, and support for the financial and practical consequences of having a long-term illness.

### Perspectives on current interventions to reduce cancer screening inequities

There was agreement that evidence-based examples of interventions within the scientific literature do not accurately reflect the breadth of inequity initiatives ongoing worldwide, and that research trials reported in the literature are only a small part of what is happening in real-world settings. For example, one co-author from the Tigerlily Foundation, which provides education, awareness, advocacy, and hands-on support to young women (aged 15-45 years) before, during, and after breast cancer, described the multitude of initiatives that the foundation uses to increase screening in different populations, that they design with local people and focus on the specific needs of the groups. They have run free drive-in movie-screenings in areas of economic deprivation where there are low rates of screening uptake among the population and provide mammography screening in mobile units at the event as well as giving gift bags to attendees that include educational information about cancer and screening or encouraging them to sign up for a mammogram. Others gave examples of similar ongoing initiatives that use activities that people find interesting or fun and integrate them with steps to increase screening uptake, whether that involves helping them to sign up for screening appointment or providing health counselling or information etc.

There was opinion that current and past interventions that aim to reduce cancer screening inequity can sometimes be limited if they use a single “one-size-fits-all” approach for all participants (for example, providing the same culturally tailored health information to all participants) whereas individually tailored interventions are likely to be more effective because they account for the person’s personal beliefs, knowledge, behaviors, and preferences. The authors felt that interventions that used one-on-one education or counselling from a lay health worker or a healthcare professional were important but also recognized the value of peer support in group-based interventions. Although there are only a few examples in the scientific literature of digital, interactive interventions that provide personally tailored approaches to address barriers to screening, it was thought that more attention should be made to develop, improve, and evaluate these methods, especially as they have the potential to be highly scalable and can be used remotely while targeting large groups of people. An individualized approach is important to grab people’s attention but may also be more effective because it can be personalized, not just to a person’s characteristics in terms of age, sex, and culture, but also to their pre-existing levels of knowledge, health literacy and beliefs, and can be delivered according to their likes and preferences. They also have the potential to be integrated into, for example, social media campaigns (discussed below) and to be linked to digital healthcare systems (for example, by improving options to digitally book screening and receive reminders from healthcare professionals etc).

### The use of digital tools and social media to reach the unreachable

One underlying theme of discussion was the need to find ways to reach unreachable people, such as individuals who never seek advice from healthcare professionals and never present to primary care clinics, perhaps due to lack of trust in authorities or medical treatment and healthcare. Many of the current examples of interventions in the scientific literature require some form of initial communication between target individuals and healthcare professionals, and for participants to be known and traceable by the people initiating the intervention. The challenge is to find ways to reach those people who never seek care from GPs and do not respond to screening reminders via letter or phone. The patient advocates emphasized the importance of understanding the target group and reaching out to them in places that they are familiar with, using techniques that resonate with them. For example, in the case of young minority women, social media outlets can be useful ways to reach them with campaigns and information about the importance of screening. There is a need to understand each target group in terms of how they use technology and social media, where they look for news or information, and then think about how these outlets can be used to bring their attention to the importance of screening or to find ways to integrate screening interventions within different technologies. Some individuals specifically expressed how it is important to not only provide health information in specific channels dedicated to health but to incorporate interventions in, for example, marketing campaigns. Although social media, websites, and applications that are specifically focused on health are important, people who seek health information there are already empowered to seek such material and, therefore, the challenge is to try to communicate with people who do not use those resources and reach them through other means, possibly with less overt techniques. One author gave the example of partnering with skincare and beauty marketing campaigns targeted to young minority women, and to integrate cancer screening interventions by, for example, raising awareness about the importance of breast cancer screening within those campaigns or addressing common barriers to screening in these women. It is important to reach out to people before they get cancer and targeting widely-used products, such as beauty and hygiene items, can reach a wider spectrum of people and their families. It was noted, however, that this approach might have better success for certain cancers, such as breast or cervical cancer, whereas it might be difficult for colorectal cancers, as some of the experts reported that there were a limited number of companies that want to be associated with this disease area. In general, a growing and stronger presence, use of, and engaged effort via the key digital platforms that the target audience uses would be helpful. Thus, this will aid in understanding not just the target audience themselves, but also how to interact with confidence in their preferred information channels in a meaningful way that balances well with other information sources and influencers [57].

### Importance of involving lay persons, patients, and survivors

Several authors felt that one of the most effective ways to reduce cancer screening inequities are programs to train individuals in the community and to subsequently work with them to hold local events or strategies that help empower individuals to undergo screening. They highlighted the importance of healthcare professionals and researchers to interact with patient organizations and then to engage target communities in culturally relevant ways that focus on their needs and preferences. Local role models can be used as ambassadors to promote screening, and lay health workers can be effective in health education, counselling, and supporting individuals. There was recognition that large community-based interventions may be costly and practically challenging, but participants felt that this was one of the most essential components needed for effective interventions to increase screening uptake. Many of the examples in the scientific literature come from specific groups such as ethnic minority groups in the USA, but the participants felt that already existing interventions that use community lay health advisors can be adapted to different cultural and economic settings, even in different counties, if the essential design of the intervention involves advocates from the target group from the start. The importance of peer-to-peer education and support was agreed but there were also proposals to create interventions that target the relatives of individuals. For example, it was highlighted that in some cases an individual can be empowered by their spouse, partner, or children and, therefore, involving them may be an important strategy to increase screening uptake.

### Empowerment

A theme that emerged during the discussion was the need to empower individuals rather than force or push them into screening programs or interventions to increase uptake. Some individuals feel that they are being told what to do by healthcare professionals, whereas people often prefer to have shared decision making between themselves and their healthcare provider, or to feel that they have control and independence to make their own decisions. Therefore, there is a need to consider an individual’s attitude towards healthcare and health systems and try to lift them from one empowerment level to another. Sometimes this can be direct but, in some cases, such individuals need to be approached in a respectful and engaging way, rather than an overtly obvious intervention that has a clear goal to increase screening uptake, which may deter them. Such examples include the previously mentioned events or activities (e.g., free movie screenings), peer coaches, and toolkits that encourage local people from the target group to attend, and to use those occasions to inform and empower individuals through various means.

### Policy and system barriers

It is important to differentiate between individual patient behaviors and broader health inequities, such as structural and systemic factors that create differential access to screening. Though individual change is relevant, there is also a wider importance of policy and system changes to tackle cancer screening inequities. Examples of interventions from the scientific literature often put focus on the individuals themselves by attempts to change their knowledge and choices about cancer screening with the aim of altering their behavior. However, this does not take into account the vast array of problems in terms of issues with healthcare systems and structures that contribute to health inequity in cancer screening (e.g., little or lack of access to care services for some individuals, challenges related to low health literacy, systemic racism etc) and a lack of clear policies and screening guidelines. One author gave the example of screening guidelines, which are sometimes not clear about which age a woman should start having regular mammography screening or that these differ between countries and regions. They highlighted how, in some places, younger women have difficulties obtaining screening, and thus changes are also needed at the system level. Others reiterated the breadth of logistical and financial barriers to screening that some people face, which cannot be addressed through interventions only aimed at empowering the individual. For example, if a person does not go for screening because they are unable to miss a work shift, other methods are needed to help improve screening rates, such as encouraging companies to add workplace protection, like being given specific time off to attend medical screening appointments. There was agreement that policies that protect employees from losing their jobs or wages are an essential component that needs to be addressed alongside empowering individuals and increasing health literacy. Addressing fragmentation within the healthcare system was also mentioned; screening is often planned and conducted from different parts of the healthcare system depending on the cancer type, yet some elements could be integrated to make it easier for people to complete screening within all oncological areas. For example, could screening for breast and cervical cancer be done at the same time to reduce some of the temporal, financial, and logistics barriers that some women face? Could different screening services collaborate to provide information and support to patients about the other types of screening? For instance, if an individual appears for cervical cancer screening, they could be reminded of, and referred to, colorectal cancer screening. It was suggested that integration and defragmentation of healthcare systems needed to be addressed to help tackle health inequity. There was also discussion about the fragmentation of patient advocacy within Europe, and the need to build a patient advocacy program into the European Union and provide legislative and practical tools to enable patient advocacy groups to have a more powerful impact.

### Essential components for designing interventions

Two of the most essential components for designing effective interventions are to ask the target population what they want and what works for them, while including professional patient advocacy groups in the co-creation of interventions at all stages of design, communication, implementation, and evaluation. Another fundamental component (discussed in more detail later) is to involve a multitude of stakeholders to implement a screening initiative (such as physicians, nurses, local organizations, health workers, digital agencies, local governments and policy makers, and patients, survivors, and representatives of the target audience) who, together, can identify the best approaches to apply in specific healthcare contexts. They can address the different potential challenges and how to address them. There was some discordance, however, on the methods needed to assess whether interventions work. Some authors felt that controlled research trials that focus only on screening uptake as an outcome can miss important elements, including feasibility and accessibility.

### The role of patients and patient advocacy organizations

Patients and patient advocacy groups are vital for helping to develop effective interventions that have positive outcomes. Indeed, previous interventions may have been unsuccessful due to not involving such people. The scientific literature has many examples of interventions that have been designed by healthcare professionals and researchers without the involvement of patients and lay persons, despite them being an essential part of the process of understanding how to better reach underserved populations. Patients and trained lay persons can be involved in a multitude of ways, both in the development and delivery of interventions to increase screening uptake. From the development perspective, multiple stakeholders should be engaged to provide a comprehensive strategy that is tailored to the specific target group in terms of cultural characteristics and health literacy levels, and with consideration of all the barriers that may be underlying low screening uptake in this population. Patient and advocacy groups are a fundamental source of such information and are trusted sources, in addition to having lived experience. This, combined with clinical experience and research-based evidence, can help to co-create strategies that have higher chance of success. Further, if individuals are aware that interventions to increase screening uptake have been designed by (or in collaboration with) patients, survivors, and advocacy groups, this may increase credibility and help to reassure people within the target group that the intervention is credible or relevant to them and their peers. From the delivery perspective, the involvement of patients and advocacy groups can help improve participation and adherence. For example, patients and survivors can become ambassadors for screening and help to encourage their peers to complete screening.

## Discussion

In this article, we have presented the perspectives of patients, patient advocates, and citizens on the current issues relating to cancer screening inequities in terms of developing and implementing interventions to reduce inequity and improve screening rates. The next steps are to consider future directions and priorities. The COVID-19 pandemic has created problems in access to screening and may potentially have created larger, or even new sources of health inequity. Thus, future interventions would likely be able to improve their designs by including lessons from the pandemic, for example, how screening services were affected, particularly in groups that often face inequity. The development of digital interventions that do not need to be delivered face to face would be an important supplement, especially interactive ones that can be tailored to the individual and have the potential to be highly scalable. However, the inclusion of digital methods needs not to be at the expense of all other efforts; it can provide useful alternatives, with added value proposition for some individuals. Digital interventions, however, can often also be quite tricky for healthcare professionals to integrate into normal practice so incorporating them only as additional resources is preferable. It is vital to always account for people who do not have access or skills to manage digital interventions and, instead, to reach out to them in places that are relevant to them.

It is important to follow guidelines for developing and evaluating complex interventions in the future. For example the UK Medical Research Council’s framework [58] outlines different phases (identify intervention, develop intervention, implementation, feasibility, evaluation) and specifies that at each phase a core element is to ask” how can diverse stakeholder perspectives be included in the research?” Indeed, a core theme that was raised within this perspectives article was the early involvement of patients, survivors, and advocacy groups in the development of interventions, and their continual participation in all stages of development, implementation, and assessment. In terms of evaluation, the success of intervention may be measured in different ways, not just in terms of screening completion, but also considering cost, scalability, how it is accepted by the target population, and whether it can be transferable to different populations that face health inequity from diverse contexts.

There are some limitations to discuss. The current paper provides the opinions of a small group of people and may not accurately reflect all perspectives. Notably, although we included health equity research experts, we focused mainly on patient advocacy groups and health empowerment organizations. We did not include any people responsible for the planning or delivery of screening services, for example. However, our aim was to focus on the perspectives of citizens and lay persons and those affected by cancer screening inequities, to underline views that are often not highlighted. Future efforts should focus on creating multi-stakeholder collaborations that include a wide range of actors from healthcare professionals to policy makers, researchers, patients, and industry experts. Further, the paper describes the views of the authors through semi-structured discussions, but specific qualitative methods could be utilized for future efforts to gain a broader perspective on a wider range of topics. A further limitation is that health inequity can differ hugely between countries, especially regarding health systems and screening services. We included individuals from North America and Europe, which have different healthcare infrastructures, but acknowledge that the perspectives described do not relate to other world locations or settings. However, as much of the previous research on screening interventions was based in USA, we felt it was important to include viewpoints from participants from this country as well as Europe.

In conclusion, cancer screening inequity remains a relevant issue and interventions to address this currently do not adequately solve the problem, especially from the perspective of patients, survivors, and lay persons. Several core pathways should be focused on in relation to designing and implementing interventions: advancing individually tailored interventions; digital tools and social media; peer-based approaches; empowerment; addressing policy and system barriers; better design of interventions; and collaboration, including the involvement of patients and patient advocacy organizations.

## Data Availability

Not applicable.

## References

[1] Global Health Europe. Inequity and inequality in Health 2009. https://globalhealtheurope.org/values/inequity-and-inequality-in-health/

[2] Siegel RL, Miller KD, Jemal A (2019). Cancer statistics, 2019. CA Cancer J Clin.

[3] Hayes L, Adams J, McCallum I, Forrest L, Hidajat M, White M, Sharp L (2021). Age-related and socioeconomic inequalities in timeliness of referral and start of treatment in colorectal cancer: a population-based analysis. J Epidemiol Community Health.

[4] Klein J, von dem Knesebeck O (2015). Socioeconomic inequalities in prostate cancer survival: a review of the evidence and explanatory factors. Soc Sci Med.

[5] Martins T, Hamilton W, Ukoumunne OC (2013). Ethnic inequalities in time to diagnosis of cancer: a systematic review. BMC Fam Pract.

[6] Forrest LF, Adams J, Wareham H, Rubin G, White M (2013). Socioeconomic inequalities in lung cancer treatment: systematic review and meta-analysis. PLoS Med.

[7] Konradsen AA, Lund CM, Vistisen KK, Albieri V, Dalton SO, Nielsen DL (2020). The influence of socioeconomic position on adjuvant treatment of stage III colon cancer: a systematic review and meta-analysis. Acta Oncol.

[8] Rollet Q, Tron L, De Mil R, Launoy G, Guillaume É (2021). Contextual factors associated with cancer screening uptake: a systematic review of observational studies. Prev Med.

[9] Mosquera I, Mendizabal N, Martín U, Bacigalupe A, Aldasoro E, Portillo I (2020). Inequalities in participation in colorectal cancer screening programmes: a systematic review. Eur J Pub Health.

[10] Unanue-Arza S, Solís-Ibinagagoitia M, Díaz-Seoane M, Mosquera-Metcalfe I, Idigoras I, Bilbao I, Portillo I (2021). Inequalities and risk factors related to non-participation in colorectal cancer screening programmes: a systematic review. Eur J Pub Health.

[11] Schueler KM, Chu PW, Smith-Bindman R (2008). Factors associated with mammography utilization: a systematic quantitative review of the literature. J Women's Health (Larchmt).

[12] Ahmed AT, Welch BT, Brinjikji W, Farah WH, Henrichsen TL, Murad MH, Knudsen JM (2017). Racial disparities in screening mammography in the United States: a systematic review and Meta-analysis. J Am Coll Radiol.

[13] Consedine NS, Tuck NL, Ragin CR, Spencer BA (2015). Beyond the black box: a systematic review of breast, prostate, colorectal, and cervical screening among native and immigrant African-descent Caribbean populations. J Immigr Minor Health.

[14] Johnson CE, Mues KE, Mayne SL, Kiblawi AN (2008). Cervical cancer screening among immigrants and ethnic minorities: a systematic review using the health belief model. J Low Genit Tract Dis.

[15] Jun J, Nan X (2018). Determinants of Cancer screening disparities among Asian Americans: a systematic review of public health surveys. J Cancer Educ.

[16] Kelly PJ, Allison M, Ramaswamy M (2018). Cervical cancer screening among incarcerated women. PLoS One.

[17] Ceres M, Quinn GP, Loscalzo M, Rice D (2018). Cancer screening considerations and Cancer screening uptake for lesbian, gay, bisexual, and transgender persons. Semin Oncol Nurs.

[18] Spence AB, Levy ME, Monroe A, Castel A, Timpone J, Horberg M, Adams-Campbell L, Kumar P (2021). Cancer incidence and Cancer screening practices among a cohort of persons receiving HIV Care in Washington, DC. J Community Health.

[19] Hart JT (1971). The inverse care law. Lancet.

[20] Landy R, Pesola F, Castañón A, Sasieni P (2016). Impact of cervical screening on cervical cancer mortality: estimation using stage-specific results from a nested case-control study. Br J Cancer.

[21] Fitzpatrick-Lewis D, Ali MU, Warren R, Kenny M, Sherifali D, Raina P (2016). Screening for colorectal Cancer: a systematic review and Meta-analysis. Clin Colorectal Cancer.

[22] Nelson HD, Fu R, Cantor A, Pappas M, Daeges M, Humphrey L (2016). Effectiveness of breast Cancer screening: systematic review and Meta-analysis to update the 2009 U.S. preventive services task force recommendation. Ann Intern Med.

[23] Otto SJ, Fracheboud J, Looman CW, Broeders MJ, Boer R, Hendriks JH, Verbeek AL, de Koning HJ (2003). Initiation of population-based mammography screening in Dutch municipalities and effect on breast-cancer mortality: a systematic review. Lancet.

[24] Lau J, Lim TZ, Jianlin Wong G, Tan KK (2020). The health belief model and colorectal cancer screening in the general population: a systematic review. Prev Med Rep.

[25] Rogers CR, Mitchell JA, Franta GJ, Foster MJ, Shires D (2017). Masculinity, racism, social support, and colorectal Cancer screening uptake among African American men: a systematic review. Am J Mens Health.

[26] Martínez-González NA, Plate A, Senn O, Markun S, Rosemann T, Neuner-Jehle S (2018). Shared decision-making for prostate cancer screening and treatment: a systematic review of randomised controlled trials. Swiss Med Wkly.

[27] Vrinten C, Gallagher A, Waller J, Marlow LAV (2019). Cancer stigma and cancer screening attendance: a population based survey in England. BMC Cancer.

[28] Ibekwe LN, Fernández-Esquer ME, Pruitt SL, Ranjit N, Fernández ME. Racism and Cancer screening among low-income, African American women: a multilevel, longitudinal analysis of 2-1-1 Texas callers. Int J Environ Res Public Health. 2021;18. 10.3390/ijerph182111267 PMC8583140 34769784

[29] Michie S, Richardson M, Johnston M, Abraham C, Francis J, Hardeman W, Eccles MP, Cane J, Wood CE (2013). The behavior change technique taxonomy (v1) of 93 hierarchically clustered techniques: building an international consensus for the reporting of behavior change interventions. Ann Behav Med.

[30] Goldman SN, Liss DT, Brown T, Lee JY, Buchanan DR, Balsley K, Cesan A, Weil J, Garrity BH, Baker DW (2015). Comparative effectiveness of multifaceted outreach to initiate colorectal Cancer screening in community health centers: a randomized controlled trial. J Gen Intern Med.

[31] Kreuter MW, Sugg-Skinner C, Holt CL, Clark EM, Haire-Joshu D, Fu Q, Booker AC, Steger-May K, Bucholtz D (2005). Cultural tailoring for mammography and fruit and vegetable intake among low-income African-American women in urban public health centers. Prev Med.

[32] Christy SM, Davis SN, Williams KR, Zhao X, Govindaraju SK, Quinn GP, Vadaparampil ST, Lin HY, Sutton SK, Roethzeim RR (2016). A community-based trial of educational interventions with fecal immunochemical tests for colorectal cancer screening uptake among blacks in community settings. Cancer.

[33] Murphy ST, Frank LB, Chatterjee JS, Moran MB, Zhao N (2015). Amezola de Herrera P, Baezconde-Garbanati LA: comparing the relative efficacy of narrative vs nonnarrative health messages in reducing health disparities using a randomized trial. Am J Public Health.

[34] Valdez A, Napoles AM, Stewart SL, Garza A (2018). A randomized controlled trial of a cervical Cancer education intervention for Latinas delivered through interactive, Multimedia Kiosks. J Cancer Educ.

[35] Elder JP, Arredondo EM, Haughton J, Perez LG, Martínez ME. Fe en Acción/faith in action: promotion of cancer screening among churchgoing Latinas. Cancer Epidemiol Biomark Prev. 2016:25.

[36] Cuaresma CF, Sy AU, Nguyen TT, Ho RCS, Gildengorin GL, Tsoh JY, Jo AM, Tong EK, Kagawa-Singer M, Stewart SL (2018). Results of a lay health education intervention to increase colorectal cancer screening among Filipino Americans: a cluster randomized controlled trial. Cancer.

[37] O'Brien MJ, Halbert CH, Bixby R, Pimentel S, Shea JA (2010). Community health worker intervention to decrease cervical cancer disparities in Hispanic women. J Gen Intern Med.

[38] Holt CL, Litaker MS, Scarinci IC, Debnam KJ, McDavid C, McNeal SF, Eloubeidi MA, Crowther M, Bolland J, Martin MY (2013). Spiritually based intervention to increase colorectal cancer screening among African Americans: screening and theory-based outcomes from a randomized trial. Health Educ Behav.

[39] Jandorf L, Bursac Z, Pulley L, Trevino M, Castillo A, Erwin DO (2008). Breast and cervical cancer screening among Latinas attending culturally specific educational programs. Prog Community Health Partnersh.

[40] De Mil R, Guillaume E, Guittet L, Dejardin O, Bouvier V, Pornet C, Christophe V, Notari A, Delattre-Massy H, De Seze C (2018). Cost-effectiveness analysis of a navigation program for colorectal Cancer screening to reduce social health inequalities: a French cluster randomized controlled trial. Value Health.

[41] Tanjasiri SP, Mouttapa M, Sablan-Santos L, Weiss JW, Chavarria A, Lacsamana JD, May VT, Quitugua L, Tupua M, Schmidt-Vaivao D (2019). Design and outcomes of a community trial to increase pap testing in Pacific islander women. Cancer Epidemiol Biomark Prev.

[42] Mehta SJ, Reitz C, Niewood T, Volpp KG, Asch DA (2021). Effect of behavioral economic incentives for colorectal Cancer screening in a randomized trial. Clin Gastroenterol Hepatol.

[43] Mehta SJ, Oyalowo A, Reitz C, Dean O, McAuliffe T, Asch DA, Doubeni CA. Text messaging and lottery incentive to improve colorectal cancer screening outreach at a community health center: a randomized controlled trial. Prev Med Rep. 2020:19. 10.1016/j.pmedr.2020.101114 PMC7251946 32477853

[44] Moscicki AB, Chang C, Vangala S, Zhou X, Elashoff DA, Dehlendorf C, Sawaya GF, Kuppermann M, Duron Y, Wyand FL (2021). Effect of 2 interventions on cervical Cancer screening guideline adherence. Am J Prev Med.

[45] Denizard-Thompson NM, Miller DP, Snavely AC, Spangler JG, Case LD, Weaver KE (2020). Effect of a digital health intervention on decreasing barriers and increasing facilitators for colorectal Cancer screening in vulnerable patients. Cancer Epidemiol Biomark Prev.

[46] Rawl SM, Skinner CS, Perkins SM, Springston J, Wang HL, Russell KM, Tong Y, Gebregziabher N, Krier C, Smith-Howell E (2012). Computer-delivered tailored intervention improves colon cancer screening knowledge and health beliefs of African-Americans. Health Educ Res.

[47] Miller DP, Denizard-Thompson N, Weaver KE, Case LD, Troyer JL, Spangler JG, Lawler D, Pignone MP (2018). Effect of a digital health intervention on receipt of colorectal Cancer screening in vulnerable patients: a randomized controlled trial. Ann Intern Med.

[48] Mehta SJ, Pepe RS, Gabler NB, Kanneganti M, Reitz C, Saia C, Teel J, Asch DA, Volpp KG, Doubeni CA (2019). Effect of financial incentives on patient use of mailed colorectal Cancer screening tests: a randomized clinical trial. JAMA Netw Open.

[49] Coronado GD, Beresford SAA, McLerran D, Jimenez R, Patrick DL, Ornelas I, Bishop S, Scheel JR, Thompson B (2016). Multilevel intervention raises Latina participation in mammography screening: findings from (sic)Fortaleza Latina!. Cancer Epidemiol Biomark Prev.

[50] Ahmed NU, Haber G, Semenya KA, Hargreaves MK (2010). Randomized controlled trial of mammography intervention in insured very low-income women. Cancer Epidemiol Biomark Prev.

[51] Sadler GR, Beerman PR, Lee K, Hung J, Nguyen H, Cho J, Huang WN (2012). Promoting breast Cancer screening among Asian American women: the Asian grocery store-based Cancer education program. J Cancer Educ.

[52] West DS, Greene P, Pulley L, Kratt P, Gore S, Weiss H, Siegfried N (2004). Stepped-care, community clinic interventions to promote mammography use among low-income rural African American women. Health Educ Behav.

[53] Horne HN, Phelan DF, Pollack CE, Markakis D, Wenzel J, Ahmed S, Garza MA, Shapiro GR, Bone L, Johnson L, Ford JG (2015). Effect of patient navigation on colorectal cancer screening in a community-based randomized controlled trial of urban African American adults. Cancer Causes Control.

[54] Kreuter MW, Holmes K, Alcaraz K, Kalesan B, Rath S, Richert M, McQueen A, Caito N, Robinson L, Clark EM (2010). Comparing narrative and informational videos to increase mammography in low-income African American women. Patient Educ Couns.

[55] Jerant A, Kravitz RL, Sohler N, Fiscella K, Romero RL, Parnes B, Tancredi DJ, Aguilar-Gaxiola S, Slee C, Dvorak S (2014). Sociopsychological tailoring to address colorectal cancer screening disparities: a randomized controlled trial. Ann Fam Med.

[56] Fernández ME, Savas LS, Carmack CC, Chan W, Lairson DR, Byrd TL, Wilson KM, Arvey SR, Krasny S, Vernon SW (2015). A randomized controlled trial of two interventions to increase colorectal cancer screening among Hispanics on the Texas-Mexico border. Cancer Causes Control.

[57] Chen J, Wang Y (2021). Social media use for health purposes: systematic review. J Med Internet Res.

[58] Skivington K, Matthews L, Simpson SA, Craig P, Baird J, Blazeby JM, Boyd KA, Craig N, French DP, McIntosh E (2021). A new framework for developing and evaluating complex interventions: update of Medical Research Council guidance. BMJ.

